# Spatial attention modulates visual gamma oscillations across the human ventral stream

**DOI:** 10.1016/j.neuroimage.2017.10.069

**Published:** 2018-02-01

**Authors:** Lorenzo Magazzini, Krish D. Singh

**Affiliations:** Cardiff University Brain Research Imaging Centre, School of Psychology, Cardiff University, Cardiff CF24 4HQ, UK

**Keywords:** Visual gamma oscillations, Visuospatial attention, Ventral visual stream, Gamma peak frequency, Magnetoencephalography

## Abstract

Oscillatory synchronization in the gamma frequency range has been proposed as a neuronal mechanism to prioritize processing of relevant stimuli over competing ones. Recent studies in animals found that selective spatial attention enhanced gamma-band synchronization in high-order visual areas (V4) and increased the gamma peak frequency in V1. The existence of such mechanisms in the human visual system is yet to be fully demonstrated. In this study, we used MEG, in combination with an optimised stimulus design, to record visual gamma oscillations from human early visual cortex, while participants performed a visuospatial attention cueing task. First, we reconstructed virtual sensors in V1/V2, where gamma oscillations were strongly induced by visual stimulation alone. Second, following the results of a statistical comparison between conditions of attention, we reconstructed cortical activity also in inferior occipital-temporal regions (V4). The results indicated that gamma amplitude was modulated by spatial attention across the cortical hierarchy, both in the early visual cortex and in higher-order regions of the ventral visual pathway. In contrast, we found no evidence for an increase in the gamma peak frequency in V1/V2 with attention. The gamma response tended to peak earlier in V1/V2 than in V4 by approximately 70 ms, consistent with a feed-forward role of gamma-band activity in propagating sensory representations across the visual cortical hierarchy. Together, these findings suggest that differences in experimental design or methodology can account for the inconsistencies in previous animal and human studies. Furthermore, our results are in line with the hypothesis of enhanced gamma-band synchronization as an attentional mechanism in the human visual cortex.

## Highlights

• A visuospatial attention task is used to probe human gamma oscillations with MEG.
• Attention increases gamma amplitude in early visual cortex (V1/V2).
• Attention increases gamma amplitude in high-level areas of the ventral stream (V4).
• No evidence of increased gamma peak frequency in V1 with attention.
• Feed-forward gamma-band synchronization as an attentional mechanism in humans.

## Introduction

The ability to direct attention to selected, relevant stimuli in a visual scene is crucial to adaptive behaviour. One proposed mechanism by which visual spatial attention is implemented at the cortical level is oscillatory synchronization in the gamma frequency range (∼30–80 Hz). The action potentials of synchronized pre-synaptic neurons arrive at the post-synaptic dendrites closer in time and sum up more effectively than those from asynchronous pre-synaptic neurons, hence increasing their downstream impact. For this reason, synchronization of neuronal firing could represent a top-down attentional mechanism to prioritize processing of attended, relevant stimuli over competing, irrelevant ones (for recent reviews, see Fries, 2015, Gregoriou et al., 2015).

The evidence in support of this model comes from studies of monkey visual area V4, where local gamma-band synchronization, measured as spectral power in the local field potential (LFP; Fries et al., 2001, Taylor et al., 2005), spike-field coherence (Bichot et al., 2005, Fries et al., 2001) or spike-spike coherence of multi-unit activity (MUA; Fries et al., 2008), is consistently stronger for attended stimuli, compared to ignored stimuli. Yet, the attentional modulation of visual gamma oscillations in the primary visual cortex is less clear. One study in monkey unexpectedly found a small, but statistically significant, increase in gamma amplitude in V1 when spatial attention was directed to stimuli outside, rather than inside, the receptive field of the recorded neurons (Chalk et al., 2010). Other studies have found no obvious effects of attention on gamma amplitude in V1 (Bosman et al., 2012, Buffalo et al., 2011). One study also found that attention modulated the gamma peak frequency in V1, which was higher in response to relevant, compared to irrelevant stimuli (Bosman et al., 2012).

Across studies in humans, the effect of spatial attention on gamma-band oscillatory activity in the early visual cortex is variable and unclear. In MEG studies, the amplitude of visually induced gamma oscillations is typically reported to increase with attention in the occipital lobe contralateral to the attended hemi-field, with sources normally extending from high-order extrastriate areas to lateral occipital and parietal cortices (Bauer et al., 2012, Bauer et al., 2014, Marshall et al., 2015a, Marshall et al., 2015b, Siegel et al., 2008). In the early visual cortical areas, i.e. presumed V1/V2, gamma oscillations are often reported to be unaffected by attention (e.g., Siegel et al., 2008). Although one study reported attentional modulations of the high-frequency gamma-band response (∼60–90 Hz) in the medial visual cortex (Koelewijn et al., 2013), this frequency range is thought to reflect different neuronal mechanisms, compared to those underlying narrow-band visual gamma oscillations (Ray and Maunsell, 2011).

Overall, the effects of spatial attention on gamma oscillations in the human early visual cortex, and in particular on the spectral properties of the V1 response, remain largely unexplored. As the uncertainties in the geometry of the source distribution can be partly attributed to the choice of stimulus configuration (see Koelewijn et al., 2013), the visuospatial attention cueing paradigm used in this MEG study was designed to induce sustained visual gamma oscillations with clear sources in the contralateral visual cortex. The accurate choice of stimulus parameters, such as size (Jia et al., 2013), spatial frequency (Adjamian et al., 2004) and eccentricity (van Pelt and Fries, 2013), allowed us to record gamma responses with a clearly quantifiable spectral profile and to test the effect of attention also on the gamma peak frequency.

## Materials and methods

### Participants

Twenty healthy volunteers took part in the study (mean age, 28.6 years; range, 22–42 years; seven males; two left-handed). All participants provided informed consent and received monetary reimbursement in agreement with the guidelines of the local ethics committee. Two participants showed no measurable gamma response to visual stimulation (see Source localization) and hence they were excluded from the analysis. The eye-tracker data were not recorded in one participant due to technical difficulties (see Eye-tracker data acquisition and analysis).

### Experimental design and paradigm

**Fig. 1:**
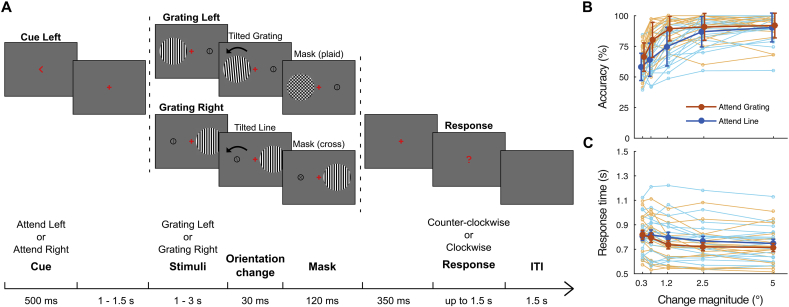
**Experimental paradigm and behavioural results. A)** Schematic illustration of the experimental paradigm. Trials started with a cue, presented for 500 m s, instructing participants which hemi-field to attend. After 1–1.5 s, two stimuli were presented, a grating and a line. After 1–3 s, the stimulus in the cued hemi-field was first presented at a tilted orientation (30 ms) and then replaced by a mask (120 ms), a plaid or a cross, depending on which stimulus was presented in the cued hemi-field. After 350 ms, participants were prompted to respond to the task by indicating whether the attended stimulus was tilted counter-clockwise or clockwise. For convenience, only the attend-left condition is illustrated in this figure, however, all four possible combinations of cue hemi-field (attend-left and attend-right) and stimulus hemi-field (grating-left and grating-right) were presented. **B)** Accuracy (i.e. percentage of correct orientation discriminations) at each magnitude of orientation change, plotted separately for each participant and also as a group average (thick lines). The error bars indicate ±1 SEM. Note that each magnitude of orientation change was increased by a factor of four, for the line stimulus (see Stimuli). **C)** The same as in B), but for response times (RTs) instead of accuracy rates.

Participants performed a visuospatial attention cueing paradigm, the task consisting of discriminating the change in orientation of the attended stimulus. The trial structure is illustrated in Fig. 1A. Each trial started with a cue, an arrow pointing either to the left or to the right side of the screen, presented centrally for 500 ms and followed by a fixation cross (0.3° of visual angle). After a jittered interval of 1–1.5 s, two stimuli (a grating and a vertical line; see Stimuli) were presented, one in the left and one in the right visual hemi-field, centred horizontally at an eccentricity of 3°. Participants were instructed to attend the stimulus in the cued hemi-field, whilst fixating centrally throughout the trial. After an unpredictable interval of 1–3 s, the attended stimulus, i.e. the one in the cued hemi-field, changed from the vertical to a tilted orientation, either clockwise or counter-clockwise. The tilted stimulus was presented for 30 ms and, to increase task difficulty, it was followed by a mask (a plaid or a cross; see Stimuli) presented for 120 ms. After a further 350 ms, a question mark prompted participants to perform a forced-choice orientation discrimination. Participants indicated whether the stimulus orientation had changed counter-clockwise or clockwise via a button-press, using the index and middle fingers of their right hand, respectively. Participants were allowed up to 1.5 s to respond and, if they had not perceived the direction of the orientation change, they were instructed to guess it. Participants were also instructed to withhold their response to any trial in which they had not complied with the task (e.g., if they had not attended the cued hemi-field). After an inter-trial interval of 1.5 s, the next trial started.

The experimental session consisted of 400 trials in total, divided into four blocks. Within each block, trials were counterbalanced across all possible combinations of four factors, namely, stimulus hemi-field (grating left vs. grating right), cued hemi-field (attend left vs. attend right), change direction (counter-clockwise vs. clockwise) and change magnitude (see Stimuli). Importantly, depending on which hemi-field was cued to attend and in which hemi-field the grating was presented, each trial fell into one of two main conditions of interest (attend grating vs. ignore grating), which were also counterbalanced. Trials were presented in pseudo-random order. To prevent habituation effects, the same combination of stimulus hemi-field and cued hemi-field was never presented for more than five times consecutively. Participants were allowed to take breaks between blocks. Each participant completed between two and four blocks (3.5 blocks on average), for a total duration of the experimental session of up to 1 h.

### Stimuli

The stimuli consisted of a grating and a line, the grating being the stimulus of interest for the analysis of visual gamma. The grating stimulus consisted of a vertical square-wave grating (maximum contrast, three cycles per degree), presented through a circular aperture with a diameter of 4°. The line stimulus consisted of a vertical black line (0.9° length, 0.05° width) enclosed in a black circle (1.1° diameter, 0.05° width). The parameters of the grating stimulus, such as size and eccentricity (4° and 3°, respectively), were designed to induce gamma oscillations with both unambiguous cortical sources (i.e. in the contralateral visual cortex; e.g., Muthukumaraswamy et al., 2009) and clearly measurable spectral properties (i.e. high-amplitude responses; see van Pelt and Fries, 2013). In contrast, the absence of high-contrast edges and the smaller size of the line stimulus were chosen to produce gamma responses of only minimal, or non-measurable, amplitude. This prevented the gamma response to the grating from being contaminated by sources in the other hemisphere, as would have happened, for example, if gratings were presented in both visual hemi-fields. Therefore, by carefully designing the psychophysical properties of both the relevant and the irrelevant stimulus, we were able to obtain uncontaminated gamma responses to the grating, whilst preserving the behavioural relevance of both stimuli to the attention task.

The difficulty of the task was varied on a trial-by-trial basis, with five possible magnitudes of orientation change increasing logarithmically from 0.3° to 5° to the vertical. To account for the different properties of the two stimuli and based on behavioural piloting, the magnitudes of orientation change of the line stimulus were increased by a factor of four compared to the grating stimulus (i.e. orientation change from 1.2° to 20° to the vertical). To increase task difficulty and hence engage participants further in the allocation of spatial attention, the orientation change was backward masked. The grating was masked by a plaid and the line was masked by a cross, both masks being presented at a tilted orientation of 45° to the vertical.

Stimuli were displayed on a gamma-corrected Mitsubishi Diamond Pro 2070 CRT monitor placed at a viewing distance of 2 m. The refresh rate was 100 Hz. Stimuli were programmed in Matlab (The Mathworks) using the Psychophysics Toolbox (Kleiner et al., 2007).

### Eye-tracker data acquisition and analysis

To monitor eye movements, monocular recordings were obtained from the right eye with an iViewX MEG250 eye-tracker (SensoMotoric Instruments) in nineteen out of twenty participants (see Participants). The video camera, operating at a sampling rate of 250 Hz, was positioned at a distance of 120 cm in front of the participant, with an infrared light placed 60 cm to the right of the camera. The gaze direction was determined based on the position of the pupil. The system was initially calibrated before the beginning of the first experimental block and then recalibrated between blocks if the head position had changed after the break.

The eye-tracker data were analysed to identify and exclude trials in which the eye gaze position deviated from the central fixation. In principle, eye gaze position or eye movements could differ between conditions, depending on which hemi-field is cued to attend. If so, any difference between the visual gamma response to attended and ignored gratings could be a spurious result of an associated difference in stimulus eccentricity (van Pelt and Fries, 2013), rather than a true effect of attention. In particular, if participants were to move their gaze towards the cued hemi-field, the eccentricity of the grating would decrease when the grating is attended, compared to when it is ignored. As such, the analysis explained below ensured that eye gaze position did not differ when participants attended the left or the right hemi-field and, consequently, towards or away from the grating (both for left- and right-presented gratings; see counterbalancing procedures in Experimental design and paradigm). Thus, we ruled out the possibility that differences in visual gamma could arise from differences in stimulus eccentricity.

The eye-tracker data analysis was performed using the EYE-EEG extension (Dimigen et al., 2011) of EEGLAB (Delorme and Makeig, 2004) and custom Matlab scripts. The raw data were cut into 2–2.5 s epochs, from cue offset (between −1 and −1.5 s) to 1 s around stimulus onset. The same pre-processing parameters were applied separately to the X and Y coordinates of gaze position, with the horizontal component being the one of interest in the analysis, for the reasons explained above. First, the data were demeaned based on the median position within each epoch. The median horizontal gaze position within this epoch was not significantly different between attend-left and attend-right trials, in any of the participants (mean *t* = −0.26 across participants). Second, short segments of missing data caused by blinks or temporary signal loss were reconstructed by linear interpolation. Third, high-frequency noise was suppressed by smoothing the data with a moving average over a window of 10 data samples. Finally, the epochs were shortened to include only the time-range in which the visual gamma response could be affected, i.e. 0–1 s around stimulus onset (see Source localization). Trials were excluded based on either deviations of horizontal gaze position from fixation or excessive horizontal eye movements (e.g., saccades) using a threshold of 2.5 standard deviations. Additionally, the horizontal gaze position was compared between cue conditions (attend left vs. attend right), within each participant. For this purpose, trials surviving artefact rejection were first pooled according to their condition and then contrasted with unpaired t-tests. This resulted in no significant difference in eye gaze position of right vs. left cue conditions, in any of the participants (mean *t* = −0.44 across participants). Altogether, therefore, these procedures ensured that the eccentricity of the gratings did not differ, because of fixation or eye movements, when they were presented in the attended or ignored hemi-field.

### MEG data acquisition and analysis

#### Data acquisition

The MEG recordings were performed using a 275-channel axial gradiometer CTF system (VSM MedTech), located inside a magnetically shielded room. The data were acquired continuously, with a sampling rate of 1200 Hz (low-pass filtered online at 300 Hz). An additional 29 reference channels were recorded for noise cancellation purposes and the primary sensors were analysed as synthetic third-order gradiometers (Vrba and Robinson, 2001). Three electromagnetic coils were placed on three fiduciary locations (nasion, left and right pre-auricular) and their position relative to the MEG sensors was recorded continuously during each experimental block.

#### MEG/MRI co-registration

An anatomical MR image (1-mm isotropic, T1-weighted FSPGR) acquired with a 3.0 T MRI scanner (General Electric) was available for each participant. For source-localization purposes, the anatomical MRI and the MEG data were co-registered by marking the voxels on the MR image corresponding to the position of the three fiducial coils (see Data acquisition).

#### MEG data pre-processing

For each participant, the data were concatenated over experimental blocks and the median head position was used as reference position for the entire dataset. The continuous dataset was then cut into epochs (±1.5 s around stimulus onset), the epochs were visually inspected and trials containing gross artefacts (e.g., muscular activity) were excluded. The position of the head within and between blocks was also visually inspected, by concatenating the continuous head position data over trials. Trials were excluded if, at any time within the trial, the distance of any of the coils from the reference position exceeded a threshold. This threshold, for the maximum distance of the head from the reference position, was defined individually for each participant (mean threshold 4.65 mm, range 2.5–7.5 mm), based on the amount of head motion.

#### Source localization

Source analysis was performed in Matlab, using the FieldTrip toolbox (Oostenveld et al., 2011). In order to reconstruct oscillatory activity at brain locations directly comparable across participants, 1) the MNI template brain was divided into a 5-mm grid with isotropic voxel resolution, 2) the individual anatomical MRI was warped to the template MRI and 3) the inverse transformation matrix was used to warp the template grid onto an individual grid for each participant. Sensor leadfields were calculated using a semi-realistic volume conduction model based on the individual anatomy (Nolte, 2003). The temporal evolution of source activation at each location in the brain was estimated using a linearly constrained minimum variance (LCMV) beamformer algorithm (Van Veen et al., 1997), with the optimal dipole orientation at each voxel estimated using singular value decomposition (SVD).

To localize the sources of visual gamma oscillations in each hemisphere optimally, the beamformer weights were calculated separately for left- and right-presented gratings. For each participant, trials were combined according to the stimulus hemi-field (left-grating or right-grating trials) and irrespective of the attention condition (both attend-grating and ignore-grating trials). To compute the weights, the covariance matrices were calculated on a time-range from −1 to 1 s around stimulus onset, between 30 and 70 Hz. The peak voxel in each hemisphere was then identified by selecting the voxel of greatest increase in gamma power (30–70 Hz), measured as percentage change between stimulus (0.3–1 s) and baseline (−0.7–0 s). The use of two separate sets of weights allowed for optimal localization of the gamma source in each hemisphere. Yet, when reconstructing the virtual sensor data (see Source reconstruction), the same weights were used to reconstruct trials of both conditions, within each hemisphere, thereby allowing the responses to attended and ignored gratings to be compared.

To compare the spatial localization of the visual gamma response to attend-grating and ignore-grating trials, the difference between the two conditions was quantified as a percentage change at each voxel location. In line with the previous source localization procedure, gamma power (30–70 Hz) was estimated during the stimulus epoch (0.3–1 s) and contrasted between attended and ignored gratings. This procedure was performed separately for left- and right-presented gratings, using the two sets of optimised weights, as described above.

#### Statistical analysis at the source level

The consistency of the visual gamma response across participants was tested statistically using a non-parametric cluster-based permutation approach, which controls for multiple comparisons across voxels (Maris and Oostenveld, 2007, Singh et al., 2003). First, the estimates of gamma power (30–70 Hz) in the baseline (−0.7–0 s) and stimulus (0.3–1 s) epochs were contrasted with paired-sample t-tests across participants at each voxel location. Second, significant t-statistics (p < 0.05) were grouped into clusters based on spatial adjacency and the t-values summed within clusters to produce a cluster-level statistic. Third, the maximum cluster-level statistic was measured in each of 10,000 Monte Carlo permutations, yielding a non-parametric null distribution that was then used to calculate the p-value of the clusters observed in the original data.

This method was applied using two different approaches. First, to compare the response to attended and ignored gratings in each hemisphere, gamma power (30–70 Hz) in the stimulus epoch (0.3–1 s) was contrasted between attend-grating and ignore-grating conditions, separately for left- and right-presented gratings. Second, to test for any sources of visual gamma insensitive to the stimulus hemi-field, the beamformer weights were re-computed after pooling trials across all conditions (calculating the covariance matrix from −1 to 1 s, between 30 and 70 Hz) and gamma power (30–70 Hz; 0.3–1 s) was contrasted between the two conditions of attention, irrespective of the grating hemi-field. In both procedures, the number of trials was equalized between conditions by random sub-sampling. The reader should note that the latter approach, in which trials are pooled across stimulus hemi-fields, should be avoided. The analysis was performed to demonstrate how the use of inappropriate analysis pipelines can lead to biased results. However, since this is neither relevant to our conclusions, nor central to our discussion, we have included these results in the Supplementary Material, rather than in the main text.

#### Source reconstruction

To analyse the effect of attention on the spectral properties of gamma oscillations in the early visual cortex (i.e. V1/V2; see Visual gamma sources in left and right visual cortex irrespective of attention), virtual sensors were reconstructed, individually for each participant and separately for each of the two peak voxel locations, by multiplying the sensor-level data by the corresponding set of optimised weights. The reconstructed single-trial time-series were first combined between left- and right-hemisphere virtual sensors and then sorted between attend-grating and ignore-grating trials. The effect of attention was analysed statistically both in the time-frequency (see Time-frequency analysis and statistics) and in the frequency domain (see Spectral analysis).

To investigate the time-course of gamma activity in downstream regions and based on the results of the statistical comparison between attention conditions (see Attentional modulation of visual gamma sources in left and right visual cortices), virtual sensors were reconstructed also in higher-order visual cortex. For this purpose, target locations were identified within the V4 complex (Bartels and Zeki, 2000) by selecting the voxel in the fusiform gyrus that was closest to the observed group-level peak t-statistic, separately in the left (MNI coordinates: [-38 -65 -15]) and in the right hemisphere (MNI coordinates: [38 -45 -10]). To remove the effect of possible spatial leakage between V1/V2 and V4, the raw virtual sensor time-series were first orthogonalised to remove zero-lag correlation (Colclough et al., 2015).

#### Time-frequency analysis and statistics

To investigate the spectral evolution of the visual gamma response over time, the virtual sensor data were represented in the time-frequency domain. For this purpose, the orthogonalised time-series from −1.5–1.5 s were bandpass-filtered at each frequency between 4 and 100 Hz, in steps of 0.5 Hz (8 Hz bandpass, 3rd order Butterworth filter) and the amplitude envelope of the analytic signal (Matlab function *hilbert*) averaged across trials (e.g., Muthukumaraswamy et al., 2010). The time-frequency maps were calculated separately for attend-grating and ignore-grating trials. A non-parametric cluster-based permutation test was then used to compare the two conditions statistically, whilst controlling for multiple comparisons across time and frequency bins (Maris and Oostenveld, 2007). In brief, first, the two conditions were contrasted with paired-sample t-tests across participants at each time-frequency bin (from −0.5–1 s, between 4 and 100 Hz). Second, significant t-statistics (*p* < 0.05) were grouped into clusters based on temporal and spectral adjacency and then summed within clusters to produce a cluster-level statistic. Third, the maximum cluster-level statistic was measured in each of 10,000 Monte Carlo permutations, yielding a non-parametric null distribution that was then used to calculate the p-value of the clusters observed in the original data.

To illustrate the changes in gamma power over time, the amplitude values from −0.5–1 s were averaged between 30 and 70 Hz, converted into percentage change from baseline (−0.5–0 s) and averaged across participants, separately for attend-grating and ignore-grating conditions.

#### Spectral analysis

The peak frequency and peak amplitude parameters of sustained visual gamma oscillations were calculated using a bootstrap procedure, which allowed also for inspection of data quality (Magazzini et al., 2016). Spectral analysis was performed using a Fourier method, the smoothed periodogram, separately for baseline (−0.7–0 s) and stimulus (0.3–1 s) epochs. The power spectrum was calculated as percentage change from baseline and the gamma peak frequency was measured, in the 30–70 Hz range, by averaging across 10,000 bootstrap iterations. The quality control (QC) was performed by calculating the width in frequency that was necessary to accommodate at least 50% of the bootstrap iterations around the bootstrap distribution mode. The data were considered of poor quality if less than 50% of the bootstrap iterations fell within ±1.2 Hz around the distribution mode (i.e. based on the frequency resolution of the periodogram).

### Behavioural data

The behavioural data were analysed in terms of accuracy rates, measured as percentage of correct orientation discriminations, and response times (RTs), calculated as time in seconds from the onset of the tilted stimulus (see Experimental design and paradigm). Trials with omissions were excluded from the analysis. Accuracy rates and RTs were calculated separately for attend-grating and attend-line (i.e. ignore-grating) conditions and separately for each magnitude of orientation change.

## Results

### Behavioural results

The behavioural responses were analysed in order to remove trials with omissions (mean ± SEM, 4 ± 1%; range, 0–20%) from the analysis of both the behavioural and the MEG data. Trials containing gross artefacts in the MEG data, excessive head motion, or eye movements (see MEG data pre-processing and Eye-tracker data acquisition and analysis) were also excluded from the MEG data analysis. The orientation of the tilted stimulus was reported correctly in 84 ± 3% (mean ± SEM) of the attend-grating trials and in 75 ± 3% (mean ± SEM) of the attend-line (i.e. ignore-grating) trials. The accuracy rates were clearly modulated by the magnitude of orientation change of both grating and line stimuli (Fig. 1B), demonstrating that participants complied with the task. The RTs were highly comparable between attend-grating (750 ± 5 ms, mean ± SEM) and ignore-grating trials (800 ± 5 ms, mean ± SEM) and were slightly modulated by the magnitude of orientation change (Fig. 1C).

### Source localization

The results of the source analyses reported below refer to the localization of the sustained component (0.3–1 s) of visual gamma oscillations, as opposed to the transient gamma response (0–0.3 s), which was not the focus of this investigation. Anatomical labels were defined by integrating the Automated Anatomical Labeling atlas (AAL; Tzourio-Mazoyer et al., 2002), the Anatomy Toolbox probabilistic atlas (Eickhoff et al., 2005) and the Talairach atlas (Lancaster et al., 2007). MNI coordinates are expressed in mm throughout.

#### Visual gamma sources in left and right visual cortex irrespective of attention

**Fig. 2:**
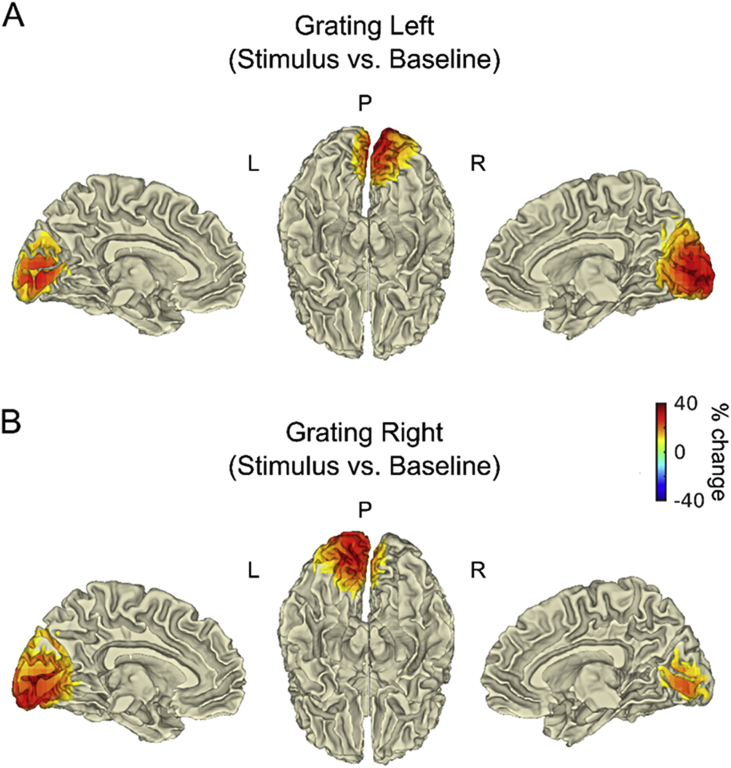
**Visual gamma response in V1/V2 irrespective of the attended hemi-field.** Beamformer source localization, projected onto the surface of an MNI template brain (left medial, bilateral inferior and right medial views, respectively). The effect of visual stimulation was measured as percentage change in gamma power (30–70 Hz) between stimulus (0.3–1 s) and baseline (−0.7–0 s), irrespective of attention and separately for gratings presented in the left (**A**) and in the right hemi-field (**B**). For visualization purposes, percentage values between ±10% were masked. P, Posterior; L, Left; R, Right.

The source analysis was performed separately for left- and right-presented gratings, by contrasting stimulus and baseline epochs irrespective of the attended hemi-field (Fig. 2). The left and right peak voxels were identified individually for each participant (see Supplementary Table 1 and Supplementary Fig. 1). Here, results refer to the average across participants. When grating stimuli were presented in the left visual hemi-field (Fig. 2A), the gamma peak response (∼38% increase from baseline) was localized to the calcarine fissure and surrounding cortex in the right hemisphere (MNI coordinates: [12 -96 -2]). When gratings were presented in the right hemi-field (Fig. 2B), the gamma peak response (∼43% increase from baseline) was localized to the calcarine fissure and surrounding cortex in the left hemisphere (MNI coordinates: [-14 -96 -2]). The peaks of these visual gamma responses are illustrated on orthogonal slices in Supplementary Fig. 2.

These results confirmed the effectiveness of the stimulus parameters (e.g., size and eccentricity of the grating) in eliciting visual gamma oscillations of clearly measurable amplitude in eighteen out of twenty participants (see Participants). Out of these eighteen participants, the right peak voxel was localized in V1 (BA17) in five and in V2 (BA18) in twelve; the left peak voxel was localized in V1 (BA17) in ten and in V2 (BA18) in eight participants. Sporadic variations in the localization of these sources (e.g., BA19 in one participants) were most likely caused by MEG-MRI co-registration errors or imperfect co-registration to the MNI template (Perry et al., 2011). Thus, to summarise, the gamma sources were unambiguously localized in the hemisphere contralateral to the stimulus hemi-field, with peaks in close proximity to V1. Hereafter, these sources will be also referred to as the “early visual cortex”.

#### Attentional modulation of visual gamma sources in left and right visual cortices

**Fig. 3:**
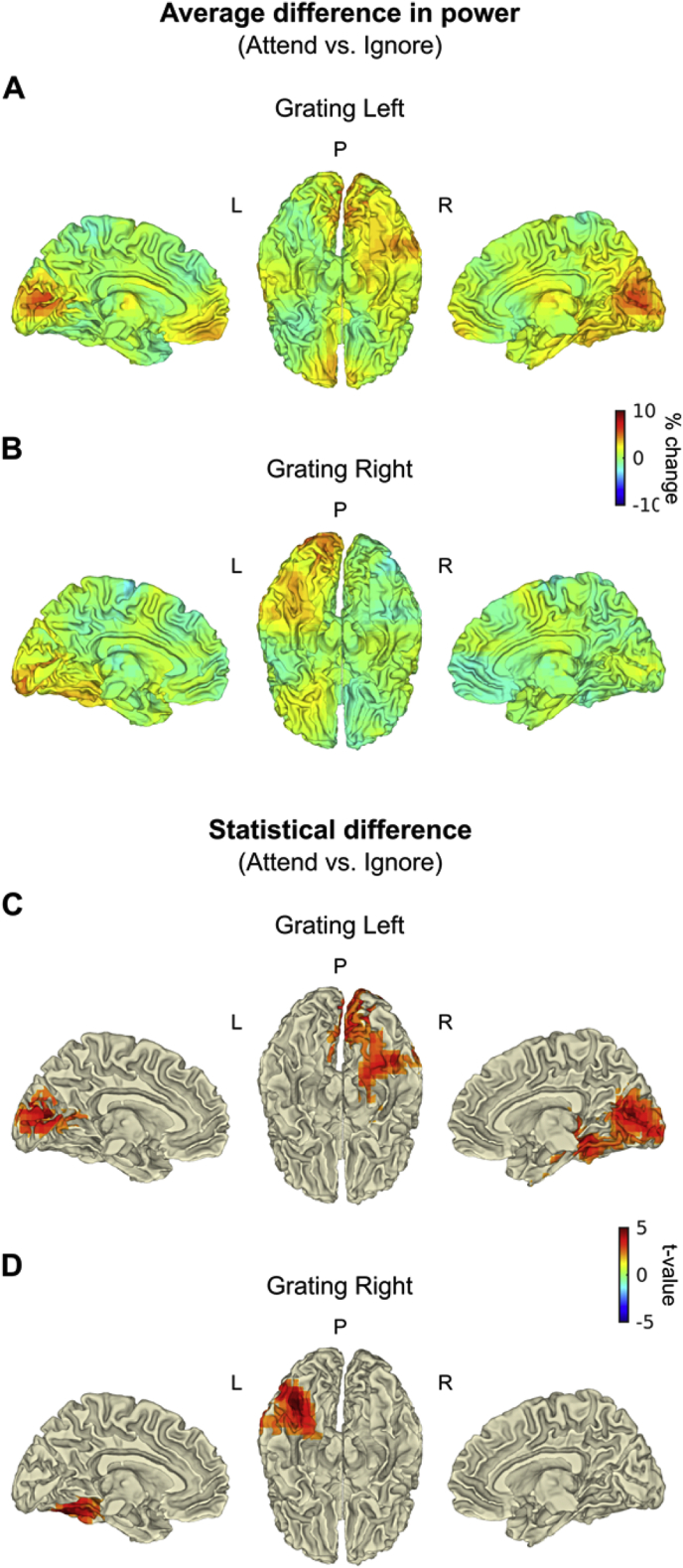
**Effect of attention at the source level. A**) and **B**) illustrate the average difference in gamma power (30–70 Hz; 0.3–1 s), calculated as a percentage change between attended and ignored gratings, separately for gratings presented in the left (A) and in the right hemi-field (B). Correspondingly, **C**) and **D**) illustrate the difference after correction for multiple comparisons, where significant paired-sample t-statistics (*p* < 0.05) were masked according to the results of a non-parametric cluster-based permutation test (*p* < 0.05, corrected). Results were projected onto the surface of an MNI template brain (left medial, bilateral inferior and right medial views). P, Posterior; L, Left; R, Right.

The effect of attention on the visual gamma sources in the left and right hemispheres was tested by comparing the response to attended and ignored gratings, separately for left- and right-presented stimuli (Supplementary Fig. 3). On average, attending the grating resulted in a 5–10% increase in gamma power in V1/V2, compared to attending the line stimulus in the opposite hemi-field. The increase in gamma power with attention peaked in the right calcarine (MNI coordinates: [22 -96 4]), when gratings were presented in the left hemi-field (Supplementary Fig. 3A), and in the left middle occipital gyrus (MNI coordinates: [-28 -96 14]), when gratings were presented in the right hemi-field (Supplementary Fig. 3B). These peak locations were ∼1 cm more lateral, compared to the peaks identified for the effect of visual stimulation irrespective of the attended hemi-field (see Visual gamma sources in left and right visual cortex irrespective of attention). Nevertheless, this increase in gamma power with attention is consistent with sources in contralateral V1/V2. These results are illustrated also in Fig. 3A and B, projected onto the brain surface.

To quantify the effect statistically at the source level, the contrast between attend-grating and ignore-grating conditions was performed using a cluster-based permutation test (see Statistical analysis at the source level), separately for left- and right-presented gratings. This analysis revealed a significant difference between conditions, for both left-presented (Fig. 3C, associated cluster: *p* = 0.014) and right-presented gratings (Fig. 3D, associated cluster: *p* = 0.041). When gratings were presented in the left hemi-field (Fig. 3C), the greatest difference within the cluster was observed in close proximity to the right inferior/middle temporal, right fusiform and right inferior occipital gyri (*t* = 5.14, MNI coordinates: [44−44 4]) and the voxels within the cluster extended to the right calcarine and surrounding cortex. When gratings were presented in the right hemi-field (Fig. 3D), the greatest differences were observed in the posterior portion of the left fusiform gyrus (*t* = 5.17, MNI coordinates: [-44 -80 -14]). Supplementary Fig. 4 illustrates these results on multiple axial slices.

### Attentional modulations in the time-frequency domain

**Fig. 4:**
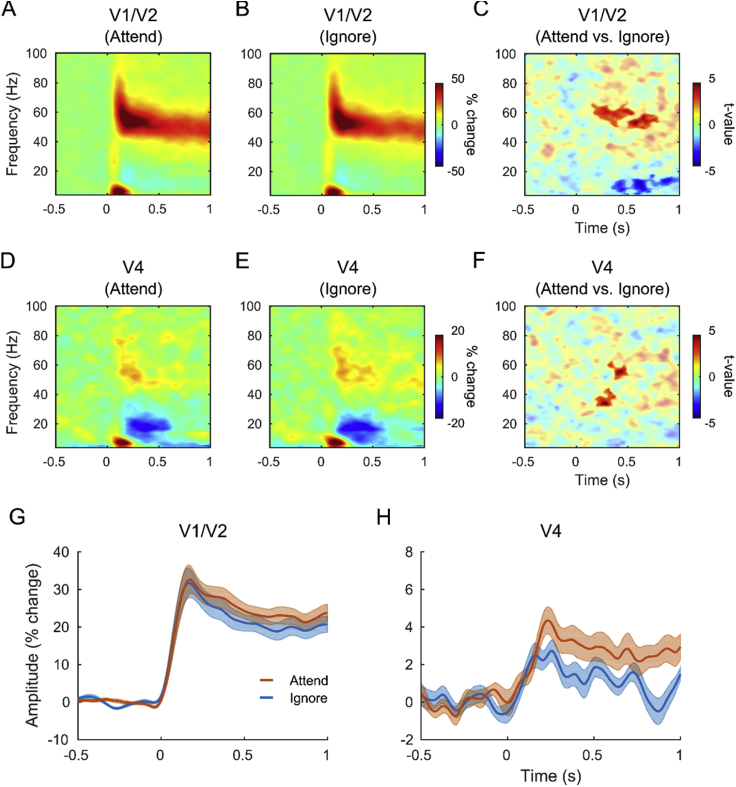
**Time-frequency analysis of the visual gamma response in V1/V2 and V4.** The virtual sensor data were reconstructed at the left and right peak voxel locations in V1/V2 (**A**, **B**, **C** and **G**) and in left and right V4 (**D**, **E**, **F** and **H**). The data were analysed separately for attended (**A**, **D**, red line in **G** and **H**) and ignored gratings (**B**, **E**, blue line in **G** and **H**) and compared statistically with a cluster-based permutation test (**C**, **F**). The clusters (*p* < 0.05, corrected) were highlighted by changing the transparency value of the colours in the plots. Note that one of the two positive clusters in **C** (500–800 ms, ∼48–60 Hz, *p* = 0.018) and one of the two clusters in **F** (∼300–500 ms, ∼50–60 Hz, *p* = 0.092) are reported for illustrative purposes only. The thick lines in **G** and **H** represent the percentage change in gamma power (averaged between 30 and 70 Hz) and the shaded areas represent ±1 SEM, across participants.

The time-frequency analysis of the virtual sensor time-series reconstructed in left and right early visual cortex (see Visual gamma sources in left and right visual cortex irrespective of attention) was performed separately for attend-grating and ignore-grating conditions (Fig. 4A and B). The effect of attention was tested using a cluster-based permutation approach, which revealed a significant difference between the response to attended and ignored gratings (Fig. 4C), with one associated positive cluster (*p* = 0.006, ∼200–600 ms, ∼50–65 Hz) and one associated negative cluster (*p* = 0.002, ∼350–1000 ms, ∼4–18 Hz). This result was followed up with a spectral analysis of the sustained visual gamma response in V1/V2 (see Spectral modulations by attention and data quality control).

The cluster-based permutation approach also revealed that the visual gamma response in V4 (Fig. 4D and E) was significantly higher in power when gratings were attended (Fig. 4F), with an associated positive cluster (*p* = 0.042, ∼200–400 ms, ∼30–45 Hz). It should be noticed, though, that the p-value reported here is statistically biased, since the V4 virtual sensors were reconstructed at the voxel location of greatest difference between conditions (see Statistical analysis at the source level). Nevertheless, this analysis qualitatively describes the oscillatory dynamics behind the statistical difference observed in the source-level contrast. The results confirm that the spectro-temporal dynamics illustrated in Fig. 4F are comparable with V4 attentional effects reported before (cf. Fig. 3 in Gregoriou et al., 2014). In addition, this increase in V4 gamma is unlikely to reflect spatial leakage from V1 for two reasons. First, the time-series were orthogonalised to remove any zero-lag correlation between the signals. Second, the evolution of gamma power (30–70 Hz) over time differed between the two regions, with the gamma response to attended gratings showing a peak earlier in V1/V2 (∼170 ms; Fig. 4G) and later in V4 (∼240 ms; Fig. 4H).

The consistency of this difference in the onset of the peak gamma response in V1/V2 and V4 was tested statistically with paired-sample t-tests across participants. For this purpose, the baselined time-frequency amplitude values were averaged between 30 and 70 Hz (see also Time-frequency analysis and statistics), individually for each participant. The time at which the increase in gamma amplitude reached a peak, relative to baseline, was identified by pooling together attend-grating and ignore-grating conditions and then compared between V1/V2 and V4 locations. As expected based on the temporal evolution of the average gamma response (in V1/V2, Fig. 4G, and in V4, Fig. 4H), the individual response peaks showed a tendency to occur earlier in V1/V2 (mean = 230 ms) than in V4 (mean = 300 ms; *t*(17) = −1.91, *p* = 0.072). As a result of the averaging procedures, the peak time of the average response differs in absolute terms from the average of the individual peak times; the latency of the peak response, however, differs on average by ∼70 m s in both analyses. In addition, it should be noticed that this result does not indicate that amplitude differences between attention conditions have an earlier onset in V1/V2 than in V4; as such, it is possible that attentional effects begin earlier in V4 than in V1/V2 (Buffalo et al., 2010, Buffalo et al., 2011).

### Spectral modulations by attention and data quality control

**Fig. 5:**
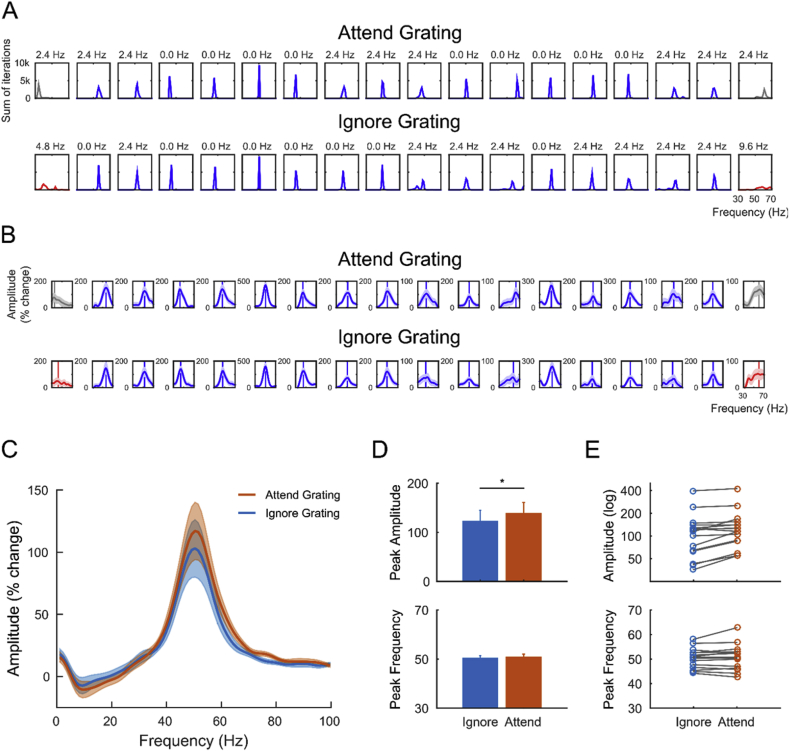
**Quality control and spectral analysis of visual gamma responses in V1/V2. A)** Individual bootstrap peak frequency distributions, calculated separately in the attend-grating and ignore-grating conditions. Poor-quality data are shown in red, list-wise rejections are shown in grey. The width in frequency necessary to accommodate 50% or more of the bootstrap iterations is shown at the top of each individual panel. **B)** Individual spectra of percentage change from baseline in the gamma frequency range (30–70 Hz). The vertical line in each individual panel indicates the bootstrap peak frequency (i.e. averaged across bootstrap iterations). The colours are the same as in A). **C)** Power spectra of percentage change from baseline in the attend-grating and ignore-grating conditions, grand-averaged across participants, separately for the two conditions. **D)** Bar graph illustrating the average peak gamma amplitude (top) and peak gamma frequency (bottom), in the two conditions. The error bars indicate +1 SEM. **E)** The same as in D), but illustrating the individual participants.

The QC analysis revealed that the spectral data in V1/V2 were generally of very good quality, with poor estimates of the gamma peak frequency in only 2 out of 36 datasets. The individual bootstrap peak frequency distributions are illustrated in Fig. 5A, separately for attend-grating and ignore-grating conditions. The individual spectra of percentage change from baseline in the gamma frequency range (30–70 Hz) are illustrated in Fig. 5B. The effect of attention on the gamma peak amplitude and peak frequency in early visual cortex was tested after exclusion of the two participants with poorly estimated gamma. Attending to the grating resulted in visual gamma responses of significantly higher amplitude (*t*(15) = 4.04, *p* = 0.001), compared to attending to the line stimulus in the opposite hemi-field. On the contrary, no significant effect of attention on the gamma peak frequency was observed (*t*(15) = 0.94, *p* = 0.36). The same pattern of results was found when all eighteen participants were included in the analysis, for both gamma amplitude (*t*(17) = 4.61, *p* = 0.0002) and gamma frequency (*t*(17) = −0.14, *p* = 0.89).

To test whether the change in peak frequency co-varied with the change in gamma amplitude in V1/V2 (see Discussion), we calculated the difference in peak amplitude and peak frequency between attended and ignored stimuli and correlated the two measures across participants. There was no evidence of a significant linear relationship between the two variables (*r* = 0.16, *p* = 0.55).

After visual inspection of the data, we tested the hypothesis that attention could modulate the so-called “centre of mass” or “spectral centroid” of the power spectra, rather than the gamma peak frequency. This measure differs from the peak frequency in that it weighs frequency (30–70 Hz) by its power across the gamma spectrum (for a more detailed explanation, see Lozano-Soldevilla et al., 2014). The result of a paired-sample *t*-test revealed a tendency for the centroid of the response to attended gratings (mean = 51.3 Hz) to be higher than the centroid of the response to ignored gratings (mean = 50.5 Hz; *t*(15) = 1.95, *p* = 0.070). This indicates that attention tended to shift the power spectra to higher frequencies by ∼1 Hz, on average, without producing an evident change in the peak frequency of the response.

## Discussion

In this study, we investigated the effects of visual spatial attention on human visual gamma oscillations. We tested the hypothesis that attention can modulate the spectral profile of the gamma response induced by visual stimulation. Despite the use of an optimal method for robust peak frequency estimation (Magazzini et al., 2016), we found no evidence of an increase in the gamma peak frequency with attention. Instead, we found that attention modulated the amplitude of sustained visual gamma oscillations in V1/V2, as well as in V4, and shifted the centroid of the power spectra in V1/V2 towards higher frequencies by ∼1 Hz on average. Together, these findings can help reconcile the inconsistent results of previous research in both animals and humans.

By combining a beamformer approach to source localization with careful design of the visual stimulus properties, we were able to record gamma oscillations from the early visual cortex contralateral to the hemi-field in which a grating stimulus was presented (Fig. 2). As expected based on evidence from previous studies in humans (e.g., Hoogenboom et al., 2006, Muthukumaraswamy et al., 2009, Swettenham et al., 2009), the individual visual gamma responses peaked in contralateral V1 or V2. The spectral analysis of the gamma response in V1/V2 revealed an increase in amplitude to attended gratings (i.e. gratings presented in the cued hemi-field) compared to ignored gratings (i.e. gratings presented contralateral to the attended hemi-field), whereas the gamma peak frequency was unaffected by attention. The spatial localization of this increase in gamma amplitude with attention was largely consistent with the sources of the gamma response induced by visual stimulation irrespective of attention (i.e. V1/V2; see Supplementary Fig. 3). Furthermore, a direct statistical comparison at source level revealed that the gamma response in higher-order visual cortices (i.e. V4) was also significantly modulated by attention (Fig. 3). By reconstructing virtual sensors in V1/V2 and in V4, after removing the effect of signal leakage with an orthogonalisation procedure (Colclough et al., 2015), these regions were confirmed as separate visual gamma sources. Thus, we can conclude that spatial attention modulates the amplitude of visual gamma oscillations across the human visual cortical hierarchy, both in the early visual cortex and in higher-order downstream regions.

These results are in line with recent theories on the role of gamma-band synchronization in attentional processing. From a theoretical perspective, post-synaptic processing stages would benefit from strongly synchronized pre-synaptic input (Engel et al., 2001). In particular, pre-synaptic neuronal groups that synchronize their firing more efficiently in response to attended stimuli can increase their post-synaptic drive, thereby facilitating the processing of attended stimulus features in downstream regions (Gregoriou et al., 2015). The attentional enhancement of gamma power in V4 observed here could thus reflect the increased efficacy by which the representation of attended stimuli in early visual cortex is propagated onto higher-order visual areas (Fries, 2015). In this view, the power increase in the early visual cortex could reflect either enhanced input from the thalamus, which would elicit the generation of higher amplitude gamma oscillations in V1 (van Kerkoerle et al., 2014), or stronger coupling between V1 and V2 (Roberts et al., 2013). Overall, this would be in line with recent theories of gamma-band activity as a mechanism for propagating sensory representations in a feedforward manner across the visual cortical hierarchy (e.g., Michalareas et al., 2016, van Kerkoerle et al., 2014).

In previous studies in animals, the strongest enhancements of gamma amplitude and gamma-band synchronization by attention have been reported in high-order visual areas, such as V4 (Bichot et al., 2005, Buffalo et al., 2011, Fries et al., 2001, Fries et al., 2008, Taylor et al., 2005). Although not to such a large extent, attentional modulations of gamma synchrony have been observed also in V2 (Buffalo et al., 2011). In V1, gamma power appears to be either decreased (Chalk et al., 2010) or unaffected by attention (Bosman et al., 2012, Buffalo et al., 2011), although the latter observation could be a result of response saturation effects due to the use of high-contrast stimuli (see Bosman et al., 2012). When comparing these results to the human MEG literature, inter-species differences, particularly those related to the generation of LFP and MEG signals (Jones, 2014), should be considered. Gamma amplitude measures, specifically, can be affected by the different spatial scale for the integration of intra-cranial LFP and scalp EEG/MEG signals (Hadjipapas et al., 2015). The magnitude of neural activity at the columnar level in V1, for example, is likely to affect spectral amplitude in the LFP signal, but not in the MEG signal. By contrast, a strong determinant of spectral power measured at the scalp level is the degree of synchrony across LFP electrodes (Musall et al., 2014). Changes in MEG gamma amplitude could thus be driven by changes in neural synchrony over large cortical patches, which would not necessarily be manifested in the LFP spectrum. As such, one possibility is that the modulation of gamma amplitude in V1/V2 observed in this study is related to synchronization over longer distances (e.g., via horizontal connections in V1) when stimuli are attended, compared to when stimuli are ignored. In the human early visual cortex, increases in gamma-band power by attention have been reported by one recent study (Koelewijn et al., 2013), though the frequency range of the effect (∼60–90 Hz) could reflect an underlying increase in neuronal firing rate, rather than in rhythmic synchronization (see Ray and Maunsell, 2011). Attentional enhancement of narrow-band gamma oscillations, instead, has been observed in early visual cortex with manipulation of attention between different sensory modalities, rather than between different locations of the visual field (Kahlbrock et al., 2012). Since other studies have not been able to implicate the early visual cortex as a source of gamma-band attentional modulations, we have identified hereafter a number of factors that could explain the (dis)similarities between our results and the existing evidence from both animals and humans.

In the human MEG literature, one study by Siegel et al. (2008), namely the first study to apply beamformer source localization in this context, investigated the effect of spatial attention on gamma oscillations induced by visual motion. The results revealed a relative increase in gamma power by attention, with extended sources in high-order visual areas that did not include the presumed V1/V2 (Siegel et al., 2008). This discrepancy with our results could be explained by the different stimulus used (i.e. random dot patterns vs. static gratings) and task required (i.e. motion direction vs. orientation discrimination) and both these factors also explain the involvement of ventral regions in our study, as opposed to the dorsal visual pathway in the study by Siegel et al. (2008). Furthermore, interpreting the sources in Siegel et al. (2008), as well as those reported by other studies (e.g., Bauer et al., 2012, Bauer et al., 2014, Marshall et al., 2015a, Marshall et al., 2015b), is complicated by the uncertainties in the source geometry underlying the response to bilateral stimuli, a configuration that may lead to self-cancellation of medial bilateral sources, when reconstructed with a beamformer (Sekihara et al., 2002; see also Koelewijn et al., 2013).

The increase in gamma amplitude in V1/V2 could also be explained by our paradigm design. The behavioural results indicated that the orientation discrimination task was harder for the line stimulus than the grating, suggesting that the task demand was high not only in the attend-grating but also in the attend-line condition. Successful allocation of spatial attention was thus required both towards and away from the grating stimulus, in order to perform the task accurately. This, together with an eccentricity of the stimulus that was unlikely to cause response saturation effects (van Pelt and Fries, 2013), may have played an important role in revealing the attentional modulation of gamma amplitude in the early visual cortex. At the same time, though, this consideration also highlights one limitation of the study, namely that the effect we refer to as an increase in amplitude with attention could in theory be driven by an underlying decrease in gamma amplitude when grating stimuli were ignored. Future studies will have the opportunity to address this concern by including an experimental condition in which attention is not cued to either hemi-field. Despite this, the interpretation of an enhancement in V1/V2 gamma by attention appears the most consistent with the literature (Buffalo et al., 2011, Kahlbrock et al., 2012).

One potential confounding factor in the interpretation of the gamma amplitude increase in V1/V2 is related to eye movements and gaze position, which, in principle, could introduce systematic differences in stimulus eccentricity between attention conditions. The visual gamma response measured with MEG is reduced for peripheral compared to foveal stimuli (van Pelt and Fries, 2013). Although this might not generalise to LFP (Lima et al., 2010) or intracranial EEG studies recording directly from the anterior-medial striate cortex (e.g., Uematsu et al., 2013), in a previous MEG study using static grating stimuli similar to ours (van Pelt and Fries, 2013), gamma amplitude was drastically reduced at an eccentricity of 6° and less so for 3° eccentricity. As estimated by van Pelt and Fries (2013), the decrease in power was accompanied by a decrease in peak frequency of ∼1 Hz/°. In our study, however, we did not observe a significant change in peak frequency with attention. In addition, the increase in gamma amplitude with attention was not significantly correlated with the change in peak frequency, across participants. For these reasons, we concluded that the procedure of trial exclusion based on changes in eye gaze position, as measured by eye-tracking, was successful in removing the possible influence of eye movements on the properties of the visual gamma response.

The second main hypothesis tested in this study concerned the peak frequency of visual gamma oscillations in V1/V2. Contrary to the effect on gamma amplitude, we found no evidence for an effect of spatial attention on the gamma peak frequency. Testing for this hypothesis was motivated by two recent studies, which reported increased gamma-band inter-areal synchronization across the visual cortical hierarchy (between V1 and V4) with selective spatial attention (Bosman et al., 2012, Grothe et al., 2012). In particular, Bosman et al. (2012) found that the gamma peak frequency in V1 was increased in response to relevant, compared to irrelevant stimuli. This suggested that the modulation of gamma frequency by top-down attentional mechanisms could serve to enhance the impact of selected upstream neurons (e.g., those in the V1 retinotopic space that represent the attended part of the visual field) on downstream neuronal groups (Cannon et al., 2014, Fries, 2015). Crucially, the shift to a higher gamma frequency with attention is thought to occur only when more than one neuronal group in V1 compete for the influence on the same neuronal group in a downstream area, such as V4 (Fries, 2015). Hence, the difference in experimental paradigm could explain why this effect was not observed here. In the study by Bosman et al. (2012; see also Grothe et al., 2012), monkeys were presented with two visual stimuli, each activating a separate recording site in V1 and the same site in V4. Due to the technical limitations of MEG, however, it would be hard to achieve a stimulus configuration that can both activate two separate, unambiguous sites in V1 and produce gamma responses of measurable amplitude in humans. Additionally, although separate activations could be achieved by stimulating both hemispheres, the use of bilateral grating stimuli would have introduced possible source cancellation problems, which, as discussed above, may have obscured the gamma response in the early visual cortex. Overall, therefore, the absence of competition among different V1 neuronal groups could well explain the lack of evidence of an increase in peak frequency with attention.

In light of the different methodologies used in animal and human electrophysiology, the discrepancy between the result by Bosman et al. (2012) and our null finding could also arise from the difference in spatial resolution itself. The oscillations recorded invasively with LFPs in monkeys are very finely resolved in space, whereas the signals recorded in humans with MEG reflect the spatial summation of synchronous neurons across larger patches of the cortical sheet (reviewed in Muthukumaraswamy, 2014). Therefore, if the gamma peak frequency measured in this study reflected the contribution of spatially distributed sources, it is possible that the modulation by attention, if any at all, was not sufficiently consistent across the visual cortex to be detected in the spatially summated response. Speculatively, it could be hypothesized that smaller neuronal groups generated spectral power at higher frequencies in response to attended stimuli, while the dominating response frequency of larger neuronal groups remained unaltered. In line with this hypothesis, the centroid (centre of mass) of the power spectra tended to higher frequencies by ∼1 Hz in response to attended stimuli, compared to ignored ones. Despite substantial inter-individual differences (see the individual spectra in Fig. 5B) this effect can be observed also in the power spectra of attended and ignored gratings, grand-averaged across participants (Fig. 5C). It is also worth noting that the gamma peak frequency differed remarkably between the two conditions of attention in some participants, although not consistently across the sample (Fig. 5E). While part of this variation is likely to reflect measurement error, the peak frequency reliability estimates obtained with our QC approach (Magazzini et al., 2016) were generally very high, within each condition. At the same time, though, the gamma peak frequency is also known to be highly repeatable within participants (Muthukumaraswamy et al., 2010, Tan et al., 2016), which leaves the individual between-condition variations observed here open to future investigations.
