# Supplementary material for: Spatial attention modulates visual gamma oscillations across the human ventral stream
Source: Neuroimage. 2018 Feb 1;166:219–29. doi: 10.1016/j.neuroimage.2017.10.069 (PMC5770333; doi:10.1016/j.neuroimage.2017.10.069)
Supplement: Online data [file mmc1.docx]

Supplementary Material

### Attentional modulation of visual gamma sources irrespective of stimulus hemi-field

To test whether any sources of gamma oscillations were modulated in a non-lateralised fashion (i.e. irrespective of the stimulus hemi-field), the effect of attention was tested statistically at source level by first pooling left-grating and right-grating trials and then contrasting the gamma response to attend-grating and ignore-grating conditions. The effect was tested statistically using a cluster-based permutation approach (see Statistical analysis at the source level). Results are illustrated in Supplementary Figure 5. The cluster with the lowest associated p-value (p = 0.078) comprised voxels confined to the early visual cortex bilaterally and the greatest difference between conditions (t = 4.99) was localized to the calcarine fissure (MNI coordinates: [0 -82 14]). In other words, V1 (BA17) showed an increase in gamma power with attention that was consistent across stimulation of both left and right hemispheres.

Importantly, it should be clarified here that this analysis approach cannot be used to infer the spatial localization of the visual gamma sources. By pooling trials across left and right grating presentations, statistical significance is biased towards those voxels showing an increase in amplitude consistently across the two hemispheres, rather than those voxels showing the strongest response to either of the two conditions. In other words, this statistical approach ‘favoured’ statistical significance for voxels that are closest to the midline and, thus, will not be discussed further.

Supplementary Table 1. Individual peak voxel coordinates.

| **Grating Left** | | | |  | **Grating Right** | | | |
| --- | --- | --- | --- | --- | --- | --- | --- | --- |
| **x** | **y** | **z** | **BA** |  | **x** | **y** | **z** | **BA** |
| 8 | -90 | 10 | 18 |  | -28 | -100 | 10 | 17 |
| 8 | -90 | 10 | 18 |  | -12 | -100 | -4 | 17 |
| 8 | -90 | -4 | 17 |  | -18 | -90 | -4 | 18 |
| 12 | -94 | -10 | 18 |  | -12 | -94 | -14 | 18 |
| 18 | -90 | 10 | 18 |  | -8 | -84 | 6 | 17 |
| 12 | -94 | 16 | 18 |  | -12 | -90 | 10 | 17 |
| 22 | -90 | 10 | 18 |  | -12 | -100 | 6 | 17 |
| 22 | -100 | 10 | 17 |  | -2 | -100 | 0 | 17 |
| 12 | -100 | -10 | 18 |  | -12 | -94 | 16 | 18 |
| 12 | -94 | 6 | 17 |  | -22 | -90 | 20 | 18 |
| 22 | -94 | -4 | 18 |  | -8 | -100 | 0 | 17 |
| 12 | -70 | 40 | 19 |  | -12 | -74 | 26 | 18 |
| 22 | -94 | 10 | 18 |  | -18 | -94 | 6 | 18 |
| 8 | -100 | 26 | 18 |  | -18 | -94 | 0 | 18 |
| 28 | -90 | -10 | 18 |  | -18 | -94 | -14 | 18 |
| 12 | -90 | 10 | 17 |  | -12 | -94 | 0 | 17 |
| 18 | -100 | 0 | 17 |  | -12 | -94 | 0 | 17 |
| 22 | -94 | 6 | 18 |  | -12 | -100 | 0 | 17 |


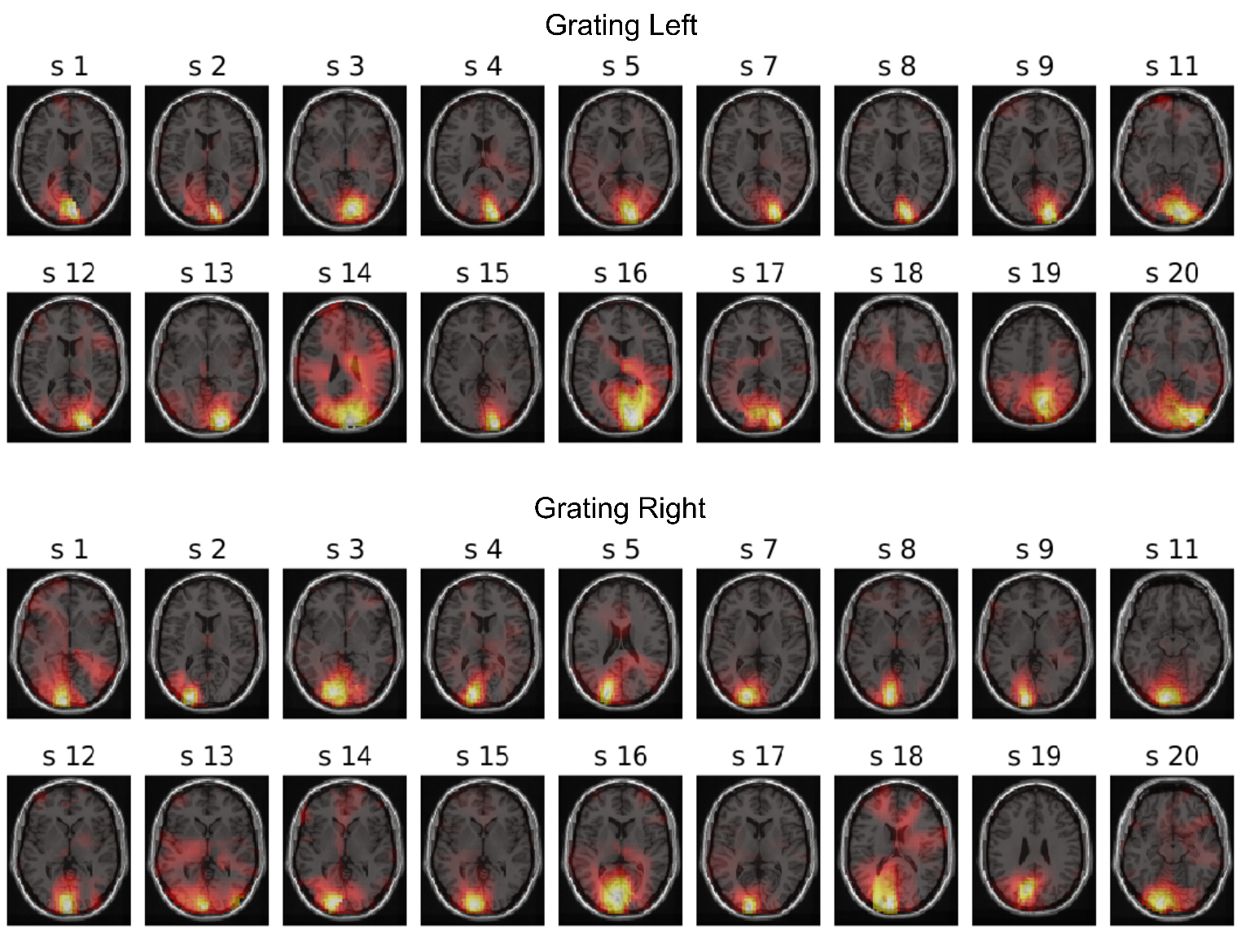


Supplementary Figure 1. Individual source localization images.

Beamformer source localization of gamma power (30–70 Hz), measured as percentage change between stimulus (0.3–1 s) and baseline (-0.7–0 s). Results were plotted on orthogonal slices of a template brain, individually for each participant, separately for gratings presented in the left (top) and right hemi-field (bottom). Trials were pooled irrespective of whether the grating was attended or ignored. For visualisation purposes, colours were scaled to the maximum in each participant and negative values were masked.


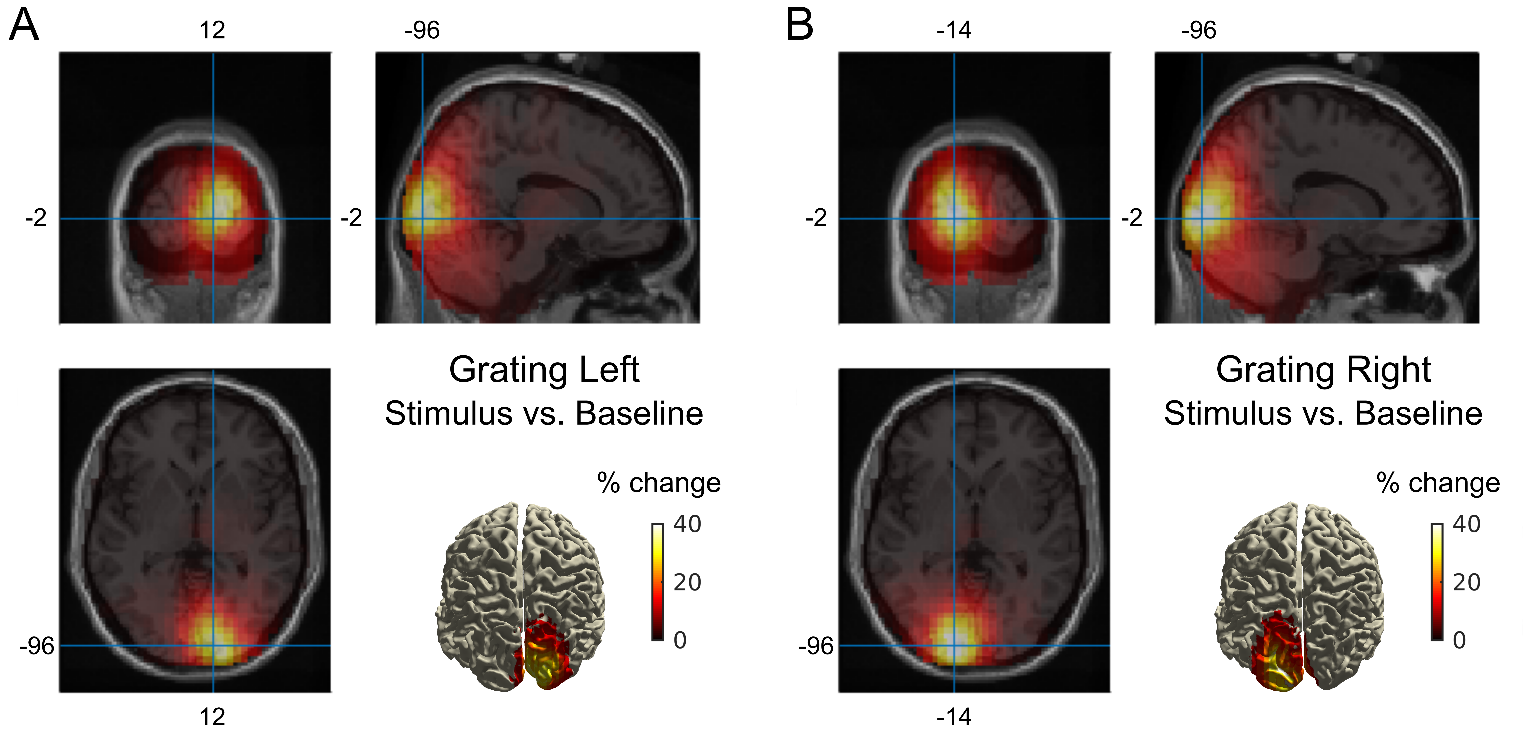


Supplementary Figure 2. Visual gamma response in V1/V2 irrespective of attention (orthogonal slices).

Beamformer source localization of gamma power (30–70 Hz), measured as percentage change between stimulus (0.3–1 s) and baseline (-0.7–0 s), for gratings presented in the left (**A**) and right hemi-field (**B**). Trials were pooled irrespective of whether the grating was attended or ignored. The individual responses were averaged across participants and plotted on orthogonal slices of a template brain. The crosshairs indicate the voxel of greatest increase in gamma power (MNI coordinates in mm). The results were also projected onto the brain surface (posterior view). For visualisation purposes, only values higher than 10% increase were projected onto the brain surface.


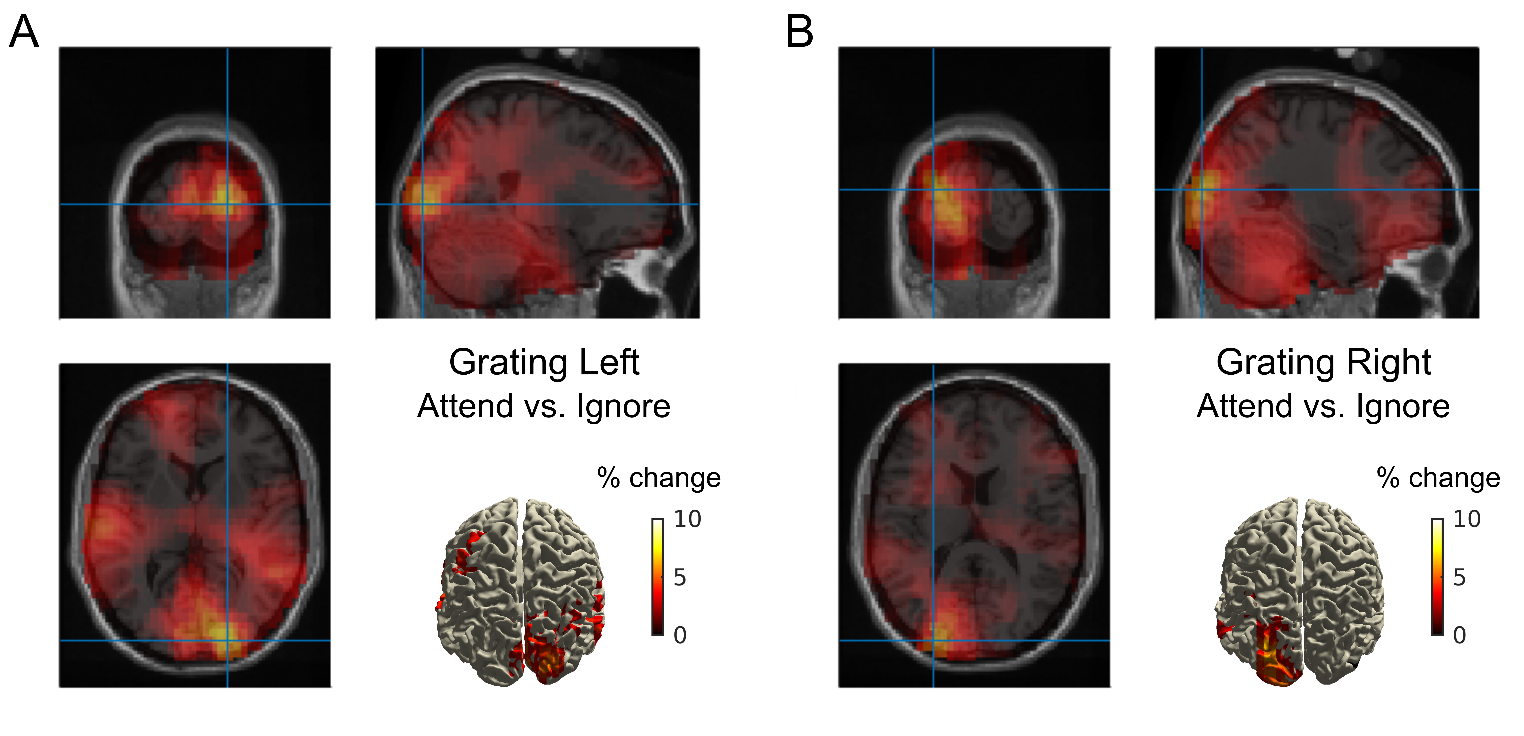


Supplementary Figure 3. Increased visual gamma response in V1/V2 with attention (orthogonal slices).

Beamformer source localization of gamma power (30–70 Hz; 0.3–1 s), measured as percentage change between attend-grating and ignore-grating conditions, for gratings presented in the left (**A**) and right (**B**) hemi-field. The individual responses were averaged across participants and plotted on orthogonal slices of an MNI template brain. The crosshairs indicate the voxel of greatest increase in gamma power. For visualisation purposes, only values higher than 3% increase were projected onto the brain surface (posterior view).


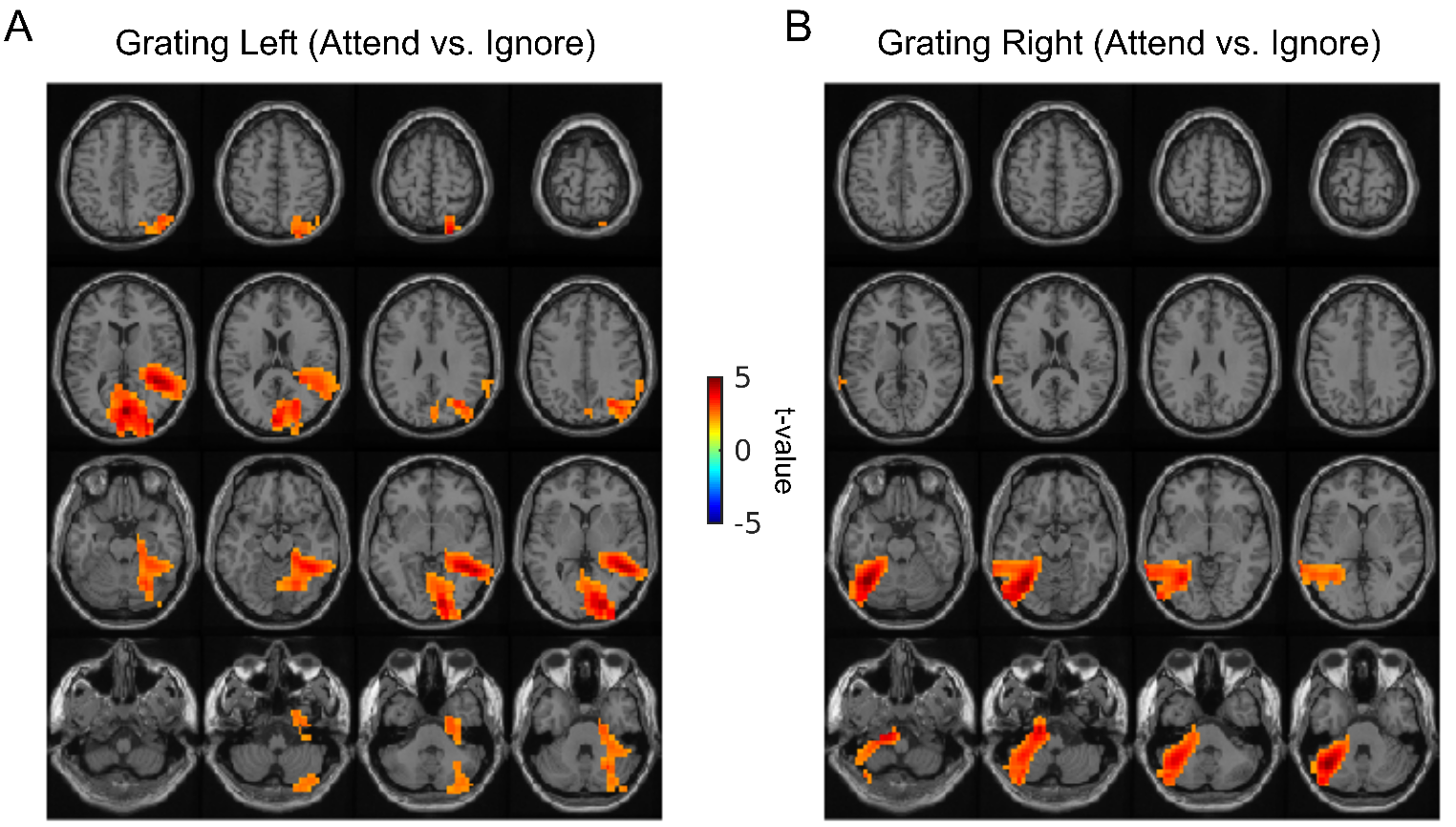


Supplementary Figure 4. Statistical analysis of the effect of attention (axial slices).

A) and B) illustrate the same results as in Figure 3C and Figure 3D, respectively, but plotted on axial slices of an MNI template brain.


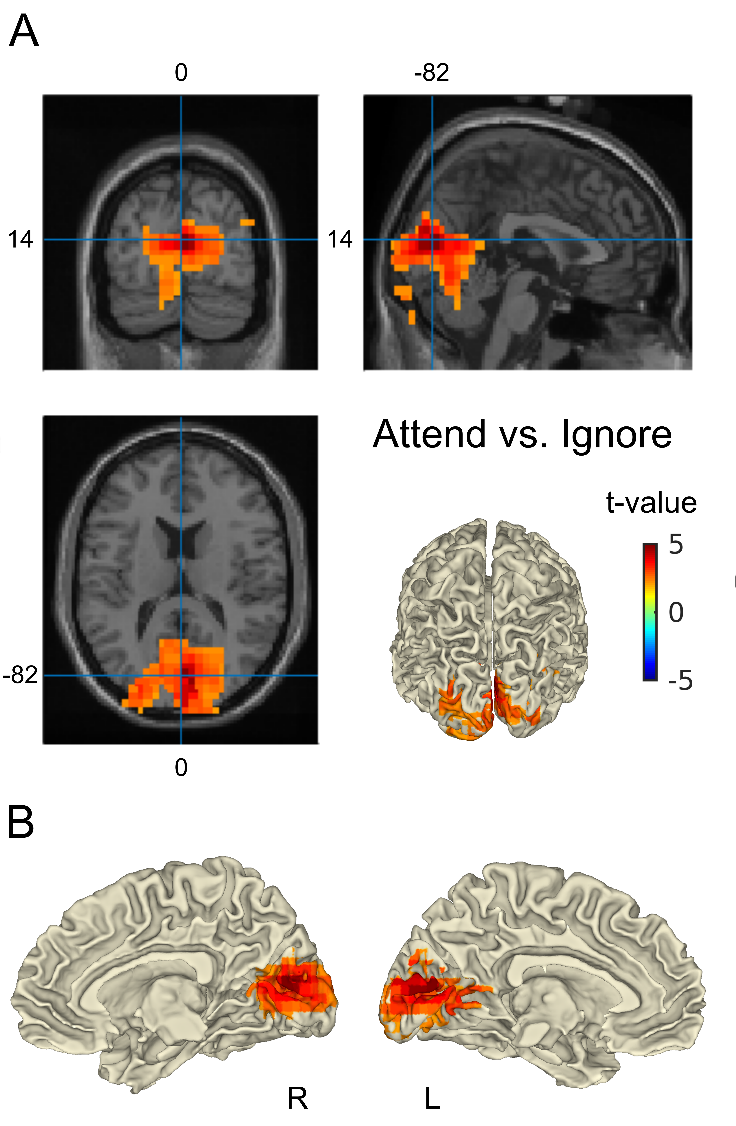


Supplementary Figure 5. Statistical analysis of the effect of attention irrespective of the grating hemi-field.

**A)** Results of the statistical analysis of gamma power (30–70 Hz; 0.3–1 s), contrasting attend-grating and ignore-grating conditions, irrespective of the grating hemi-field (i.e. with trials pooled across left- and right-presented gratings). The t-values (paired-sample t-tests) were masked according to the results of the cluster-based permutation test (*p* = 0.078, corrected). The crosshairs indicate the voxel with the largest t-value (*t* = 4.99, MNI coordinates: [0 -82 14]). In the bottom-right panel, results were projected onto the surface of an MNI template brain (posterior view). **B)** Medial views of the surface projection, right (R) and left (L) hemispheres.
